# First principles study on the structural stability, mechanical stability and optoelectronic properties of alkali-based single halide perovskite compounds XMgI_3_ (X = Li/Na): DFT insight

**DOI:** 10.1039/d4na00305e

**Published:** 2024-07-24

**Authors:** Kefyalew Wagari Guji, Tesfaye Abebe Geleta, Nabil Bouri, Victor José Ramirez Rivera

**Affiliations:** a Physics Department, Bule Hora University P. O. BOX 144, Bule Hora Ethiopia gujikefyalew@gmail.com; b Department of Agricultural Chemistry, National Taiwan University Taipei 10617 Taiwan tesfaye867@gmail.com; c Laboratory of Materials Physics and Subatomic, Faculty of Science, University Ibn Tofail BP. 133 14000 Kénitra Morocco; d Department of Physics, Jorge Basadre Grohmann National University Tacna Peru

## Abstract

Metal-halide perovskites are recognized as cutting-edge solar energy technology, boasting remarkable absorption capabilities, minimal environmental impact, and cost-effectiveness. This study delves into the structural stability, mechanical stability, and optoelectronic properties of lead-free halide perovskites, specifically XMgI_3_ (X = Li/Na), by utilizing the CASTEP and WIEN2k software along with the GGA-PBE and Tran–Blaha modified Becke–Johnson (TB-mBJ) exchange–correlation functions to compare their electronic properties. The structural and mechanical stabilities were confirmed by assessing their tolerance factor and formation energy and by evaluating their elastic constants, respectively. Using the TB-mBJ exchange–correlation potential function, the calculated indirect band gap values for LiMgI_3_ and NaMgI_3_ were 2.474 and 2.556 eV, respectively. These band gaps are suitable for solar energy harvesting due to their broad optical absorption ranging from infrared to visible light. The partial density of states and the total density of states were determined to investigate the contribution of individual atoms. Consequently, this study can guide researchers focusing on the experimental synthesis of these materials at the laboratory scale for in-depth exploration, particularly in applications such as photovoltaics and various optoelectronic devices.

## Abstract 2

Metal-halide perovskites are recognized as cutting-edge solar energy technology, boasting remarkable absorption capabilities, minimal environmental impact, and cost-effectiveness.

## Graphical abstract

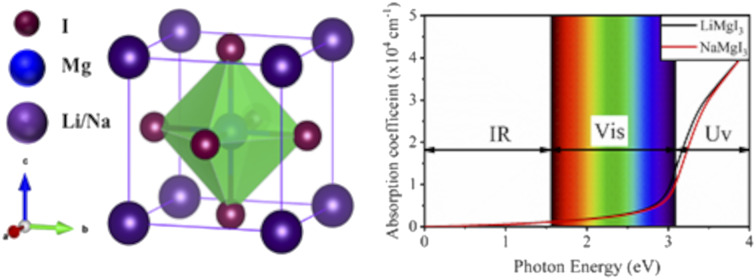

## 1. Introduction

With the increase in the world's population and economy, the demand for energy is growing.^1^ However, non-renewable energy sources such as fossil fuels employed to meet this demand are being depleted,^2,3^ and the energy generated from non-renewable energy sources is harmful to the surrounding environment due to CO_2_ emissions.^4^ Therefore, it is necessary to discover safe and reliable new energy technologies. To solve this kind of problem, perovskite materials (ABX_3_)^5^ have the characteristics of photovoltaics (PV), the most common type of solar technology,^6,7^ due to their significant properties^6,8–10^ that contribute to the production of clean energy from pollution.^11–13^ The most promising solar energy in recent years has been organic–inorganic halide perovskite (OIHP)-based PV technology, which is efficient, inexpensive, and has special optoelectronic effects.

However, the low stability of OIHPs hinders their application in the production of solar cells. For example, conventional OIHPs such as MAPbI_3_ (ref. 14) and MAPbX_3_ (X = Cl, Br, and I)^15^ have attracted considerable interest due to their high efficiency and suitability for optoelectronic applications, despite concerns regarding their instability and the toxicity associated with the presence of lead (Pb). Most of the metal halide Pb-free materials such as ZYbI_3_ (Z = Rb, Cs) have been used as perovskite materials as a result of their high efficiency.^16^ Mahmood *et al.*^16^ reported the preparation of RbYbI_3_ and CsYbI_3_ with a narrow direct band of 1.22 eV and 1.12 eV, respectively, which are considered suitable for the fabrication of solar cells.

Interestingly, Ray *et al.*^17^ proposed substituting divalent atoms (such as Ge, Sn, Pb, Mg, Ca, Sr, and Ba) for Pb to address the toxicity concerns associated with perovskite materials in photovoltaic applications. They found that Mg and Ba perovskites are unlikely to form in the cubic, tetragonal, and orthorhombic phases due to their positive formation energies. Alternatively, Ca and Sr perovskites exhibit negative formation energies with respect to the metal-iodide precursors and they possess wide band gaps, making them less favorable candidates for use in photovoltaic devices. They additionally reported that the predictive capability of a local density functional with a non-separable gradient approximation (NGA) closely resembles that of hybrid functionals in terms of band gap predictions, particularly when M in CsMI_3_ represents a p-block element such as Pb, Sn, and Ge.

The stability of perovskite materials was identified to be enhanced by introducing K and Rb atoms,^18–21^ specifically KGeX_3_ and RbGX_3_ (where X = Cl or Br). Due to their exceptional optoelectronic properties, which include a high static refractive index, low reflectivity, high absorption coefficient, and excellent stability, they have been thoroughly investigated for their possible uses in solar energy and photoelectric systems. Furthermore, they exhibit relatively low levels of toxicity. Additionally, CsGeX_3_ (X = F, Cl, and Br) perovskite has been identified as a promising candidate for optoelectronic applications owing to its exceptional electronic, optical, thermoelectric, and tunable band gap properties.^5,22,23^ Lead-free metal halide compounds XBeCl_3_ (X = Ga/Ag)^24,25^ and NaXCl_3_ (X = Be/Mg)^26^ have been investigated for application in optoelectronic devices due to their unique structural, optical, electronic, and elastic properties. GaBeCl_3_ and AgBeCl_3_ have band gaps of 3.03 eV and 3.25 eV, while NaBeCl_3_ and NaMgCl_3_ show band gaps of 4.15 eV and 4.16 eV, respectively. An increase in atomic radii corresponds to a decrease in band gap. These materials also exhibit ductility, enhancing their suitability for use in optoelectronic devices. First-principle calculations were used to comprehensively evaluate these properties.

The electrical and optical properties of different cubic lead-free halide perovskites, CsMgX_3_ (X = Cl and Br) compounds, were studied.^27^ Similar to these compounds, the cubic halide perovskite CsBX_3_ (B = Sn, Ge, X = I, Br, and Cl) compounds were reported to be suitable for optoelectronic energy devices.^28^ Furthermore, recently narrow band gap perovskite materials such as BaZrS_3_ (ref. 6) and CsYbI_3_ (ref. 16) were reported. Owing to their narrow band gap, the reported materials are highly recommended for perovskite solar cell applications due to their wide absorption spectrum, primarily covering the visible and infrared regions. However, their elements are categorized as transition metals and lanthanides, which are computationally expensive and result in the formation of compounds with a relatively high density. In the calculation of the electronic structure of transition metals, standard density functional theory (DFT) underestimates the results. Therefore, it is essential to consider additional methods such as incorporating the Hubbard–U function or hybrid function.

Caid *et al.*^29^ introduced Cs_2_CdZnCl_6_, a lead-free halide double perovskite, *via* DFT calculations. This compound exhibits mechanical stability in the cubic nonmagnetic phase and has a direct band gap of 1.43 eV. Its optical conductivity was determined to be up to 13.0 eV, showing a significant ultraviolet photoresponse. These characteristics suggest that Cs_2_CdZnCl_6_ is promising for optoelectronic applications, making it a valuable addition to materials science and technology. In another study, Caid *et al.*^30^ extensively investigated Cs_2_B′B′′Br_6_ double perovskites, revealing their potential in various technological fields. These compounds demonstrated robust stability with negative formation energies and favorable elastic constants. In the case of both BeMg and CdBe, Cs_2_B′B′′Br_6_ exhibited direct band gaps, whereas indirect band gaps were observed with CdGe, GeMg, GeZn, and MgZn. The optical property assessments highlighted the exceptional dielectric characteristics and strong light absorption in visible and UV spectra of these perovskites, making them promising candidates for solar cells and energy harvesting technologies, indicating significant potential for future energy and optoelectronic advancements.

Therefore, to address these issues, we were motivated to develop transition metal-free and lightweight compounds within alkali and alkaline-based halide perovskite materials (XMgI_3_, X = Li/Na). Our proposed materials exhibit superior optoelectronic properties compared to previous reports, particularly maintaining a narrow band gap, which enhances the photon absorption region. To the authors' knowledge, there are currently no reports available on alkali metal-based iodide perovskite compounds, namely LiMgI_3_ and NaMgI_3_, based on either DFT simulations or experimental studies. Thus, this study aimed to thoroughly investigate the mechanical stability, optoelectronic characteristics, and atomic population analysis of XMgI_3_ (X = Li/Na) using DFT and the CASTEP and WIEN2k software. This article presents XMgI_3_ (X = Li and Na) perovskite compounds as novel and inventive materials for solar cell applications that outperforms all previously investigated metal-halide perovskites.

## 2. Computational analysis

In this investigation, we applied the plane wave pseudopotential approach utilizing CASTEP software^31–34^ based on DFT principles. The electron exchange-correlation energy was computed following the Perdew–Burke–Ernzerhof (PBE) scheme within the framework of the generalized gradient approximation (GGA).^35^ We used an ultrasoft pseudopotential^36^ to describe the atomic potential. The electron configurations of the Li, Na, Mg, and I atoms are 1s^2^2s^1^, [Ne] 3s^1^, [Ne] 3s^2^, and [Kr] 5s^2^4d^10^5p^5^, respectively. For optimizing the crystal structure, we employed a plane wave cutoff energy of 700 eV and a 6 × 6 × 6 *k*-point grid in the Brillouin zone^37^ for both XMgI_3_ (X = Li/Na) compounds. Structure optimization and lattice parameter calculations were carried out using the BFGS method. The specific tolerances included a total energy of 5.0 × 10^−6^ eV per atom, self-consistent field (scf) of 5.0 × 10^−7^ eV per atom, maximum force of 0.01 eV Å^−1^, maximum stress of 0.02 GP, and maximum displacement of 5.0 × 10^−4^ Å. The elastic constants, electron localization function, and Mulliken population analysis were performed using CASTEP software. The Bader change analysis was performed using Quantum ESPRESSO.

To precisely assess the ground-state characteristics and optoelectronic properties of the materials, the full-potential linearized augmented plane wave (FP-LAPW)^38^ method within the WIEN2k code was used.^39^ The Trans–Blaha (TB-mBJ) exchange–correlation approximations were employed. Implementing the TB-mBJ function yielded a highly accurate band gap value.^40,41^ For the specified objective, the adjusted fundamental parameters were chosen as the standard configuration in the computational software, incorporating *R*_MT_ × *K*_max_ = 8, with *K*_max_ denoting the wave vector and *R*_MT_ representing the muffin-tin sphere radius. A *k*-mesh of 15 × 15 × 15 was chosen to ensure precise energy calculations. The self-consistent field method with an energy convergence threshold of 10^−4^ Ry was employed in the iteration process. The comparison of the GGA-PBE function employed in CASTEP and WIEN2k was conducted comprehensively in this study.

Moreover, to validate the significance of FP-LPAW in conjunction with the mBJ hybrid function, an analysis employing the Heyd–Scuseria–Ernzerhof 2006 functional (HSE06) within the framework of Quantum ESPRESSO (QE) was conducted.^42^ In the process of the scf calculation, a convergence threshold of 60 Ry was established for the *E*_cutoff_ wave function, and a denser *k*-mesh of 14 × 14 × 14 in the Brillouin zone was utilized. The phonon dispersion analysis was conducted through the utilization of the phonopy module integrated in the WIEN2k software. Moreover, *ab initio* molecular dynamics (AIMD) simulations were executed using the Forcite tools, wherein the NVE thermodynamic ensemble was designated to assess the thermal stability of the compounds under investigation at room temperature.

## 3. Results and discussion

### 3.1 Structural analysis

**Fig. 1:**
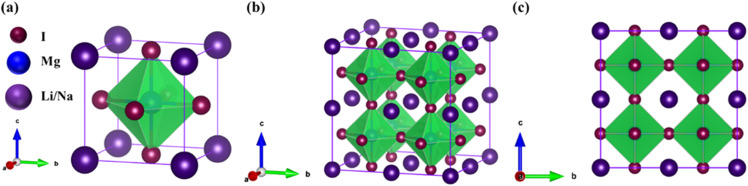
Optimized crystal structure of XMgI_3_ (X = Li/Mg) halide-based single perovskite compounds: (a) 3D unit cell, (b) 3D, and (c) 2D supercell (2 × 2 × 2). The purple-color ball represents a Li or Na atom, the blue-color ball represents an Mg atom, and the dark-purple color represents an I atom.

**Fig. 2:**
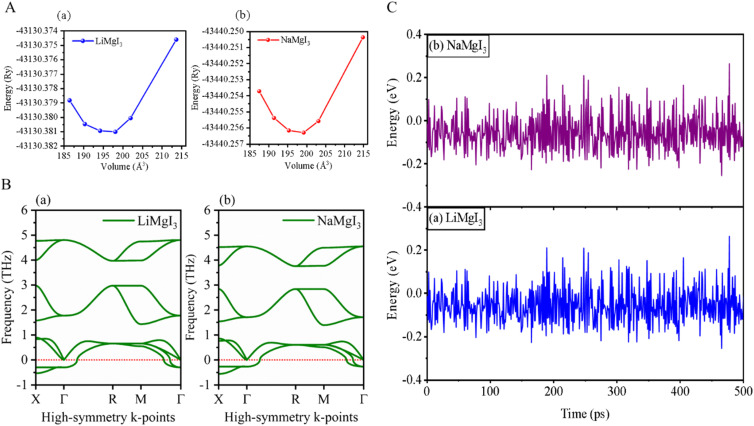
(A) Total energy with volume optimization curves, (B) phonon dispersion, and (C) thermal stability of LiMgI_3_ and the NaMgI_3_, respectively.

The investigated alkali-based single halide perovskite compounds, denoted as XMgI_3_ (X = Li/Na), exhibit a cubic ABX_3_ structure, conforming to the *Pm*3̄*m* crystallographic space group (no. 221). The cubic primitive unit cell of XMgI_3_ (where X = Li/Na) comprises five atoms. Specifically, the X atoms are positioned at the corners in the 1a Wyckoff position (0, 0, 0), the Mg atom occupies the body center in the 1b Wyckoff position (0.5, 0.5, 0.5), and the I atoms are positioned at the face center in the Wyckoff position (0.5, 0.5, 0.0). The optimized bulk crystal structure of XMgI_3_ (X = Li/Na) is given in Fig. 1. Table 1 displays the computed the lattice parameters (*a* = *b* = *c*), band gap (*E*_g_), cell volume (*V*), tolerance factor (*τ*), and formation energy (*E*_f_) values for the investigated compounds. To further confirm the structural stability, specifically the lattice parameters, of the cubic perovskite materials, we performed the optimization utilizing the CASTEP and WIEN2k code tools. The optimized lattice constants for LiMgI_3_ and NaMgI_3_ were calculated to be 5.8022/5.8297 Å and 5.8207/5.8408 Å utilizing CASTEP and WIEN2k with the corresponding cell volumes of 194.2461/198.0969 Å^3^ and 195.3316/199.2664 Å^3^, respectively, as indicated in Table 1 and Fig. 2A. The lattice parameters increased by approximately 0.47% and 0.34% for LiMgI_3_ and NaMgI_3_, respectively, while the volume increased by nearly 2% for both materials. These results indicate that their cubic structures remain stable even when optimized with different tools. Due to ionic differences between the Li and Na atoms, variations in both their lattice constants and cell volume were observed when utilizing the same tool (either CASTEP or WIEN2k).

**Table 1:** The computed optimized lattice constants (*a* = *b* = *c*), band gap (*E*_g_) using CASTEP, WIEN2k, and QE, cell volume (*V*), formation of energy (*E*_f_), and tolerance factor (*τ*) for the cubic structure of XMgI_3_ (X = Li/Na) halide single perovskite compounds and some reported data

Compounds	Lattice constants, *a* = *b* = *c* (Å)		*E*_g_ (eV)				*V* (Å^3^)			*E*_f_ (eV)	*τ*	Ref.
	CASTEP	WIEN2k	PBE-CASTEP	PBE-WIEN2k	mBJ-WIEN2k	HSE06-QE	CASTEP	WIEN2k	Δ*V* (%)			
LiMgI_3_	5.8022	5.8297	1.174	1.181	2.474	2.219	194.246	198.097	1.98	−1.8864	0.81	This study
NaMgI_3_	5.8207	5.8408	1.176	1.185	2.556	2.239	195.332	199.266	2.01	−1.9021	0.87	This study
LiMgI_3_	—	5.805	—	1.132	2.586	—	—	—	—	—		43
LiCaI_3_	—	6.1382	—	2.457	4.525	—	—	—	—	—		43
NaCaI_3_	—	6.146	—	2.527	4.600	—	—	—	—	—		43
NaMgCl_3_	—	4.940	—	—	4.16	—	—	—	—	−0.923		26
NaBeCl_3_	—	4.550	—	—	4.16	—	—	—	—	−1.023		26

The Goldschmidt tolerance factor, denoted as “*τ*”, is widely used to investigate the structural stability of ABX_3_ perovskites, giving insight to determine the compatibility of the cations within their structure, which is defined as follows:^44^1
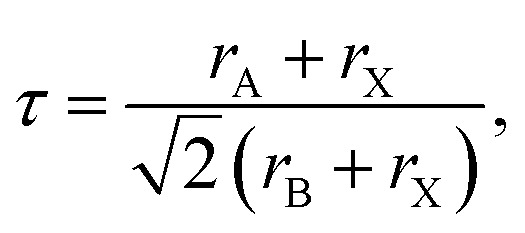
where *r*_A_, *r*_B_, and *r*_X_ are the A-site, B-site, and X-site ionic radii, respectively. In general, perovskite structures are stable in cubic structures when their tolerance factor is in the range of 0.8 < *τ* < 1.^45–48^ Alternatively, when *τ* < 0.8, the perovskite adopts an orthorhombic structure, whereas for *τ* > 1, it is categorized as having a hexagonal crystal structure.^49^ The ionic radii used in this calculation were 1.13, 1.39, 0.72, and 2.20 Å for Li, Na, Mg, and I, respectively.^47^ Thus, the calculated tolerance factors of 0.81 for LiMgI_3_ and 0.87 for NaMgI_3_ fall within the acceptable range, as listed in Table 1, resulting in the confirmation of the ideal cubic structural stability of the investigated compounds.

Furthermore, the formation energy (Δ*E*_f_) of the compounds was calculated to confirm their thermodynamic stability at room temperature using eqn (2), as follows:^50^2
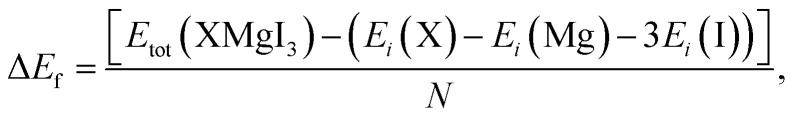
where *E*_tot_(XMgI_3_) represents the total energy of the XMgI_3_ compound, *N* is the number of atoms, and *E*_i_(*X*), *E*_i_(Mg), and *E*_i_(I) are the total energy of the individual atoms, including X (where X = Li and Na), Mg, and I, respectively. It is evident that the obtained negative values of the formation energy in Table 1 indicate the stability of the proposed perovskite compounds under ambient conditions. This information is valuable for experimentalists considering the synthesis of these materials at the laboratory scale. To the best of our knowledge, these materials have not been experimentally reported. Nevertheless, the comparable works reported using DFT, focusing on the lattice parameters and band gap, are compiled in Table 1 to validate the credibility of our study.

Furthermore, phonon dispersion calculations offers valuable insights into the dynamical stability of the materials being studied.^51^ The phonon dispersion curves were obtained by computing the phonon frequencies along the high symmetry directions (*X* → *Γ* → *R* → *M* → *Γ*) of the Brillouin zone, as illustrated in Fig. 2B. Typically, stable phonon structures exhibit real frequencies, as denoted by positive signs, whereas unstable structures are characterized by imaginary frequencies, which are indicated by negative signs. The computed phonon spectra revealed that both the LiMgI_3_ and NaMgI_3_ perovskite materials exhibit real frequencies at the gamma (*Γ*) point, confirming their dynamic stability. However, at the *X*-point in the first Brillouin zone, a less negative phonon frequency (∼−0.54 THz) was observed, suggesting localized instabilities or specific vibrational modes affecting certain directions or regions within the crystal lattice.^52,53^ Positive phonon frequencies at the *Γ*-point indicate stable vibrational modes at the center of the Brillouin zone, typically corresponding to the collective motions of the entire crystal lattice or large-scale atomic displacements. These stable modes contribute to the overall stability of the material by ensuring well-defined lattice vibrations without leading to global instabilities.

To evaluate the thermal stability of LiMgI_3_ and NaMgI_3_, *ab initio* molecular dynamics simulations (AIMD) were carried out at room temperature over a period of 500 ps, utilizing a time step of 1 fs. The results, as depicted in Fig. 2C, reveal that minimal energy fluctuations occurred during the simulation. Furthermore, both perovskite frameworks preserved their original configurations without exhibiting any discernible structural deformities. Thus, these findings undeniably confirm the stability of LiMgI_3_ and NaMgI_3_ at room temperature.^26^

### 3.2 Atomic populations analysis

Mulliken atomic population (MAP) is suitable for gaining insight into the atomic bonding (such as chemical bonds, bond overlap, and movement of charge between atoms) in compounds.^54,55^ The calculated bond populations, charges, and bond lengths are summarized in Table 2 for the XMgI_3_ (X = Li/Na) compounds. The determined positive (Li, Mg, and Na) and negative (I) charges in the XMgI_3_ (X = Li/Na) compounds, as shown in Table 2, suggest that charge is transferred from the alkali metals (Li and Na) and alkaline earth metallic (Mg) atoms to the halogen (I). In both the LiMgI_3_ and NaMgI_3_ compounds, charge transfer of 0.46 *e* and 0.28 *e* to iodine (I), respectively, was observed. Moreover, we conducted theoretical investigations into the charge-transfer mechanism utilizing Bader analysis,^56^ as depicted in Table 2. The Bader charge analysis utilizes electron density to determine the electronic charges associated with individual atoms.^57^ It helps in understanding the nature of chemical bonding and intermolecular interactions. The ability of the I atom to accept electrons from Li/Na and Mg suggests its ionization nature. Substituting Li with Na resulted in slight alterations in the Bader charges, as shown in Table 2. In the LiMgI_3_ structure, the average Bader charge on the I atoms was −0.816 *e*, while that in the NaMgI_3_ structure was −0.784 *e*. The variation observed in the charge distribution computed using Mulliken and Bader analyses likely stems from their differing methodologies. Bader analysis emphasizes the topological aspects of electron density, whereas Mulliken analysis relies on population analysis.

**Table 2:** Charge spilling parameters (%), orbital charge (*e*), atomic Mulliken charge (*e*), Bader charge (*e*), bond population, and bond length for the LiMgI_3_ and NaMgI_3_ compounds

Compound	Species	Charge spilling (%)	Atom population			Mulliken charge (*e*)	Bader charge (*e*)	Bond	Bond population	Bond length (Å)
			s	p	Total					
LiMgI_3_	Li	0.34	2.15	0.00	2.15	+0.850	+0.959	I–Li	0.04	4.095
	Mg		0.67	6.81	7.48	+0.520	+1.488	I–Mg	0.10	2.896
	I		1.88	5.58	7.46	−0.460	−0.816	I–I	−1.84	4.095
NaMgI_3_	Na	0.22	2.18	6.40	8.59	+0.410	+0.928	I–Na	0.04	4.103
	Mg		0.73	6.86	7.58	+0.420	+1.424	I–Mg	0.02	2.901
	I		1.74	5.53	7.28	−0.280	−0.784	I–I	−1.50	4.103

Additionally, Table 2 provides data on the bond populations, population ionicity, and bond lengths for the Li–I, Mg–I, Na–I, and I–I bonds within the XMgI_3_ (X = Li/Na) perovskite structure. Positive and negative values signify bonding and antibonding states, respectively, with a higher positive bond population denoting increased covalency in the bond.^58^ For example, the lower positive bond population in Li/Na–I (associated with longer bond lengths) and Mg–I (associated with shorter bond lengths) in Table 2 suggests a high level of ionic character and insignificant covalent bonding in both compounds. Additionally, the negative bond population of I–I indicates the formation of antibonding interactions.

### 3.3 Electron localization function

**Fig. 3:**
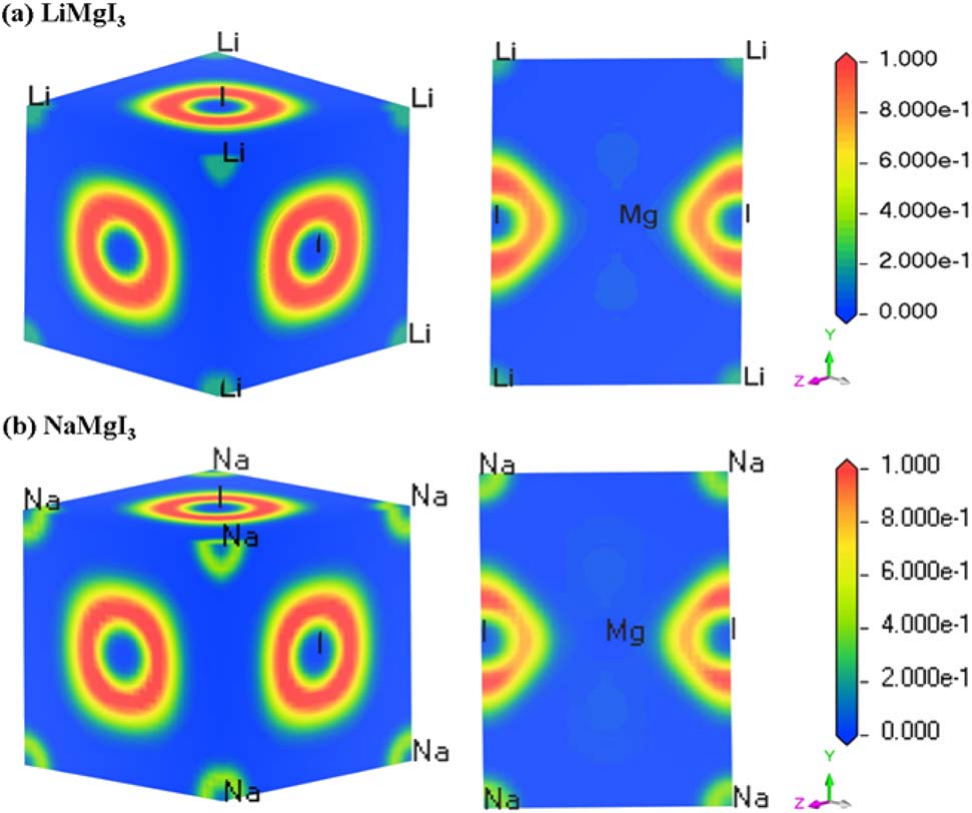
DFT-computed electron localization function (ELF) in the 3D and 110 crystal direction of (a) LiMgI_3_ and (b) NaMgI_3_ single perovskite compounds, respectively.

The electron localization function (ELF) serves as a valuable tool for analyzing the electron distribution and localization during the formation of a crystalline structure.^59^ ELF quantifies the probability of finding electrons with identical spins and fixed positions within a reference electron field, offering insights into the electron localization within the lattice space and revealing the bonding characteristics in materials. Fig. 3 shows the 3D and 110 crystal dimension ELF maps for the XMgI_3_ compounds (X = Li/Na). The right side of the ELF maps illustrates the ELF scaling, including the coordination scaling, where regions with lower electron density are depicted in blue and that with higher electron density in red. The examination of the ELF maps for the XMgI_3_ compounds revealed a heightened degree of electron localization around the I ions, which is attributed to their significant electronegativity. This observation is consistent with the findings from the Mulliken population analysis and the Bader charge analysis.

### 3.4 Elastic constants and mechanical properties

The mechanical and structural stability of materials can be determined by measuring their elastic constant (*C*_*ij*_). These measurements indicate that materials deform under stress but return to their original shape once the stress is removed.^60^ Thus, to ensure the materials' mechanical stability, we calculated *C*_*ij*_ using the finite strain theory, providing insights into their strength and response to external forces. In this investigation, we first performed elastic constant calculations, followed by determining the mechanical and structural stability parameters, as listed in Table 3. The mechanical properties of these materials include hardness, softness, compressibility, ductility, brittleness, and strength, which are vital for industrial applications. In cubical crystals, there are three independent elastic constants, *C*_11_, *C*_12_, and *C*_44_,^61^ which were calculated using the finite strain theory.^62^ According to the symmetry results, *C*_11_ = *C*_22_ = *C*_33_, *C*_12_ = *C*_23_ = *C*_13_, and *C*_44_ = *C*_55_ = *C*_66_.^63^ Alternatively, it is feasible to determine the Young's modulus (*E*), shear modulus (*G*), bulk modulus (*B*), anisotropy modulus (*A*), and Poisson's ratio (*σ*) using the elastic constants.^64–66^ In addition, the elastic constant is used to determine the mechanical stability of the compounds according to the Born stability criterion,^67^ where the crystal deformations in a compound depends on a specific criterion.^68^ In the case of the cubic phase, three independent elastic constants are required to fulfill the Born stability criterion, as given in eqn (3), to determine the stability of a compound:^69^

3 *C*_11_ > 0, *C*_44_ > 0, *C*_11_ − *C*_12_, *C*_11_ − 2*C*_12_ > 0

**Table 3:** The calculated elastic constant *C*_*ij*_ (GPa), bulk modulus *B* (GPa), shear modulus *G* (GPa), Young's modulus *E* (GPa), Poisson's ratio (*σ*), Pugh ratio (*B*/*G*), Cauchy pressure (*C*_P_ = *C*_12_ − *C*_44_) (GPa), anisotropy factor (*A*), sound velocity (V), Debye temperature *θ*_D_ (K), and melting temperature *T*_m_ (K) of XMgI_3_ (X = Li/Na) single perovskite compounds

Material property	LiMgI_3_	NaMgI_3_
*C*_11_ (GPa)	43.10	43.38
*C*_12_ (GPa)	16.06	15.93
*C*_44_ (GPa)	10.97	11.53
Bulk modulus, *B* (GPa)	25.08	25.08
Shear modulus, *G* (GPa)	11.93	12.36
Young's modulus, *E* (GPa)	30.88	31.85
Poisson's ratio, *σ*	0.29	0.29
Pugh ratio, *B*/*G*	2.10	2.03
Cauchy pressure, *C*_P_ (GPa)	5.09	4.40
Anisotropy factor (*A*)	0.81	0.84
Transverse sound velocity, *v*_t_ (m s^−1^)	1839.41	1843.22
Longitudinal sound velocity, *v*_l_ (m s^−1^)	3409.56	3379.92
Average sound velocity, *v*_m_ (m s^−1^)	2051.79	2053.93
Debye temperature, *θ*_D_ (K)	180.37	180.22
Melting temperature, *T*_m_ (K)	807.72	809.38

The elasticity of length and shape is related to the *C*_11_ and *C*_12_ constants, respectively. For a cubic structure, *C*_11_ must be considerably larger than *C*_12_.^61^ Interestingly, the estimated elastic constants for both the LiMgI_3_ and NaMgI_3_ compounds listed in Table 3 confirm they satisfy the Born stability criteria, indicating their mechanical stability. The Pugh ratio (*B*/*G*) and Cauchy pressure are useful parameters to assess a material's brittleness and ductility.^60^ In this case, a material is determined to be ductile when *B*/*G* is equal to or greater than 1.75, whereas when it is less than 1.75, the material is brittle.^70^ According to this study, the (*B*/*G*) ratios for LiMgI_3_(2.1) and NaMgI_3_(2.03) are higher than 1.75, suggesting that these materials exhibit ductile behavior, as shown in Table 3.

The Cauchy pressure for the cubic LiMgI_3_ and NaMgI_3_ determines their ductility and brittleness based on the negative and positive values of *C*_12_ − *C*_44_, as previously reported.^71^ A positive value indicates ductility, whereas a negative value suggests brittleness. In this study, the difference between *C*_12_ and *C*_44_ (Table 3) for both compounds are positive values, resulting in indicating ductility. Alternatively, the Voigt–Reuss–Hill (VRH) method is an approach used to calculate the *B*, *G*, *E*, and *σ* of the materials. The Voigt and Reuss methods are used to determine the bulk^72^ and shear^73^ moduli of materials, respectively, while Hill's^64^ method is used to calculate the average values of these properties. The Reuss bulk modulus (*B*_R_) and Voigt bulk modulus (*B*_V_) are given by eqn (4):4
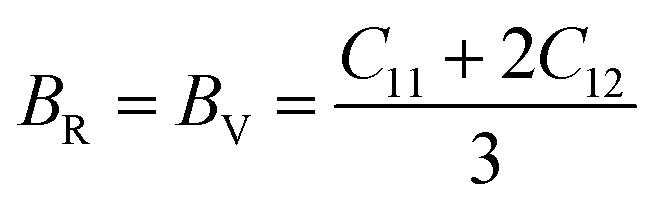


The shear modulus (*G*_V_) of a material according to Voigt^72^ is presented in eqn (5):5
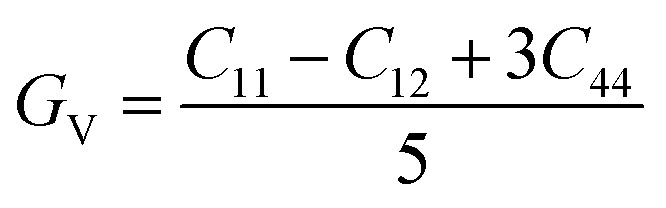
moreover, the shear modulus (*G*_R_) was calculated by Reuss using eqn (6):^73^6
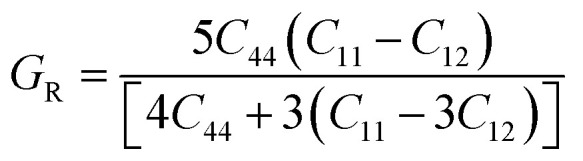
and their average value was computed by Hill,^64^ as indicated in eqn (7) and (8), respectively:7
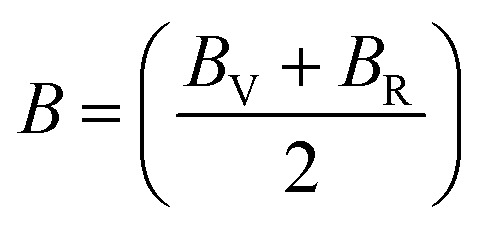
8
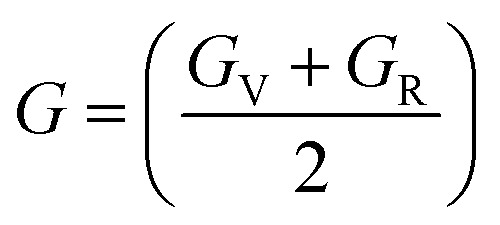


The values for *B*, *G*, and *E* are presented in Table 3. The *B* (25.08, 25.08), *G* (11.93, 12.36), and *E* (30.88, 31.85) of LiMgI_3_ and NaMgI_3_, respectively, indicate that both materials exhibit comparable resistance to changes in volume and shape.

Moreover, the observed *E* and *σ* listed in Table 3 can be calculated using the following expression, respectively:^69^9
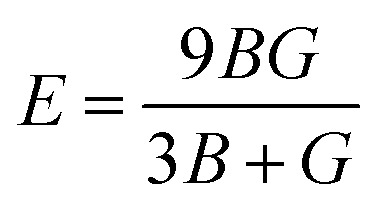
10
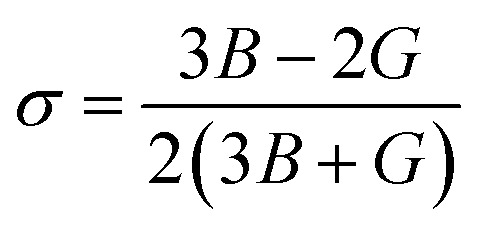


The resistance of a material, which is indicated by its stiffness, is quantified by *E*, where materials with high *E* values exhibit increased resistance. Therefore, as shown in Table 3, LiMgI_3_ and NaMgI_3_ exhibit identical and high E values, suggesting that they possess a significant level of stiffness. Alternatively, *σ*, which is limited to values between 0.25 and 0.50, determines the behavior of bond forces. A *σ* value greater than 0.26 indicates that the material is ductile, whereas a value less than 0.26 indicates that it is brittle.^74^ Hence, as indicated in Table 3, the bond force of the LiMgI_3_ and NaMgI_3_ compounds is close to the center of the above-mentioned range (0.29). As a result, both compounds exhibit a characteristic of ductility that agrees well with the results of the *B*/*G* ratio and Cauchy's pressure equation.

Moreover, the elastic anisotropy factor (*A*), which is used to determine the direction-dependent properties of a system, was computed by using eqn (11):^69^11
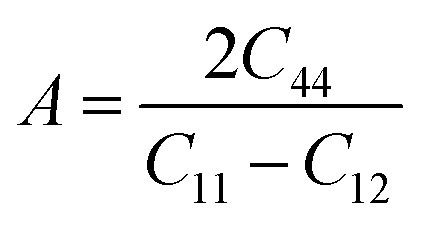


For isotropic compounds, *A* is ideally 1, indicating uniformity, whereas any deviation from 1 indicates the presence of anisotropy.^75,76^ As shown in Table 3, the computed anisotropy factors for both compounds deviated from unity (0.81 for LiMgI_3_ and 0.84 for NaMgI_3_), indicating the presence of anisotropy in these materials.

The calculation of *G* and *B* enables the determination of the transverse sound velocity, *v*_t_ (eqn (12)), and longitudinal sound velocity, *v*_l_ (eqn (13)), of XMgI_3_ (X = Li/Na) crystals, where *ρ* refers to the density of the investigated compounds as follows:^77–80^12
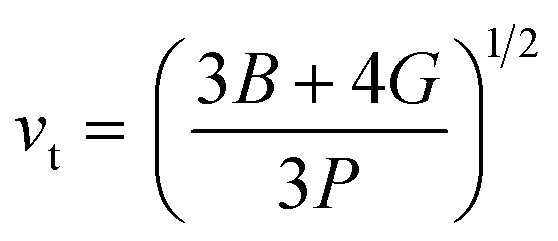
and13
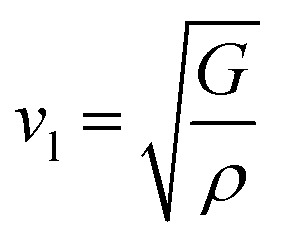


Moreover, utilizing eqn (10) and (11), eqn (12) allows the calculation of the mean sound velocity (*v*_m_), as follows:14
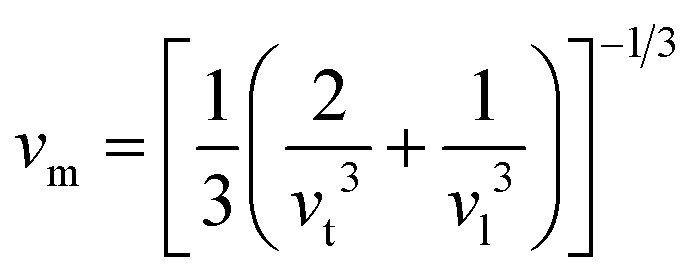


The Debye temperature (*θ*_D_) is used to determine the thermal characteristics of a material including its melting temperature and thermal expansion. Employing eqn (14), *θ*_D_ of LiMgI_3_ and NaMgI_3_ can be obtained, as expressed in eqn (15):15
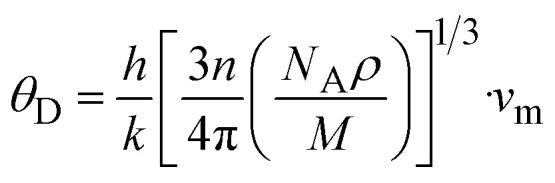
where *h* represents the Planck constant, *k* represents the Boltzmann constant, *N*_A_ denotes the Avogadro constant, *M* signifies the molecular mass, and *n* indicates the number of atoms in the compound. This calculation yielded valuable insights into the thermal properties of the investigated crystals.

The calculated values (*v*_t_, *v*_l_, and *v*_m_) and *θ*_D_ for the LiMgI_3_ and NaMgI_3_ compounds are shown in Table 3. The calculated parameters for LiMgI_3_ and NaMgI_3_ compounds approximately show similar *θ*_D_, making both compounds have similar thermal conductivity and melting temperature. The melting temperature (*T*_m_) is a crucial parameter for determining the bond characteristics and thermal conductivity of XMgI_3_ (X = Li/Na). The melting temperatures of these cubic materials, as listed in Table 3, were calculated using eqn (16):^81–83^

16 *T*_m_ = 553 + 5.91*C*_11_

As illustrated in Table 3, both LiMgI_3_ and NaMgI_3_ exhibit high and comparable melting temperatures (*T*_m_), indicating their suitability for high-temperature optoelectronic applications. Thus, the high computed melting temperatures for these compounds imply their strong bonding characteristics and excellent thermal conductivity.

### 3.5 Electronic properties

**Fig. 4:**
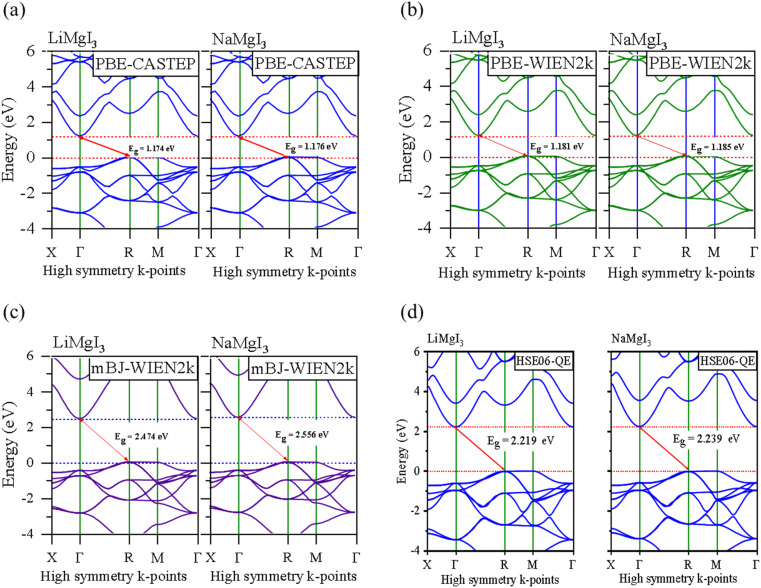
Band structures computed using (a) GGA-PBE-CASTEP, (b) GGA-PBE-WIEN2k, (c) mBJ-WIEN2k, and (d) HSE06-QE for LiMgI_3_ and NaMgI_3_, respectively. The Fermi level is set to zero.

In this section, we present the analysis of the energy dispersion and electronic states of the compounds, which determine their electronic properties. The electronic bandgap (*E*_g_) of the materials was obtained through band structure analysis, while their partial density of states (PDOS) and total density of states (TDOS) were determined, which describe the distribution of electronics at different energy levels. The electronic properties of the XMgI_3_ (X = Li/Na) single perovskite materials were investigated using the CASTEP and WIEN2k codes. Fig. 4 and Table 1 demonstrate the narrow indirect band gaps of 1.174/1.176 eV, 1.181/1.185 eV, 2.474/2.556 eV, and 2.219/2.239 eV for LiMgI_3_/NaMgI_3_, which were calculated employing PBE-CASTEP, PBE-WIEN2k, mBJ-WIEN2k, and HSE06-QE, respectively. It is clear that the standard DFT PBE function consistently underestimated the electronic structure, particularly the band gap, in both CASTEP and WIEN2k, as shown in Fig. 4a and b, respectively. However, despite this underestimation, the results suggest that the proposed compounds exhibit sufficient stability, given that they yielded similar outcomes across different computational codes. Furthermore, the mBJ correlation function (Fig. 4c) accurately predicted the band gap, which correlates well with the experimental values. This suggests that LiMgI_3_ and NaMgI_3_ have a favorable band gap, which shows their potential for use as semiconductor materials in PV devices and other optoelectronic applications. Further examination was conducted to validate the prediction by the mBJ correlation function employing the alternative robust HSE06 hybrid functional (Fig. 4d), leading to the sustained accurate correlation with the mBJ prediction. The stability and reliability of both LiMgI_3_ and NaMgI_3_ are evident from the predictions by the mBJ and HSE06 correlation functions, suggesting their suitability for future empirical investigations.

Fig. 4a–d show that both the conduction band minimum (CBM) and valence band maximum (VBM) of the LiMgI_3_ and NaMgI_3_ compounds are located at *Γ* and *R*, respectively, in the first Brillion zone, which agrees with the previous finding.^84^ The s-block VB metals are flat between the *R* and *M* points, suggesting the presence of heavy holes, whereas the p-block metals have a greater dispersed CB at the *Γ*-point.^85^

**Fig. 5:**
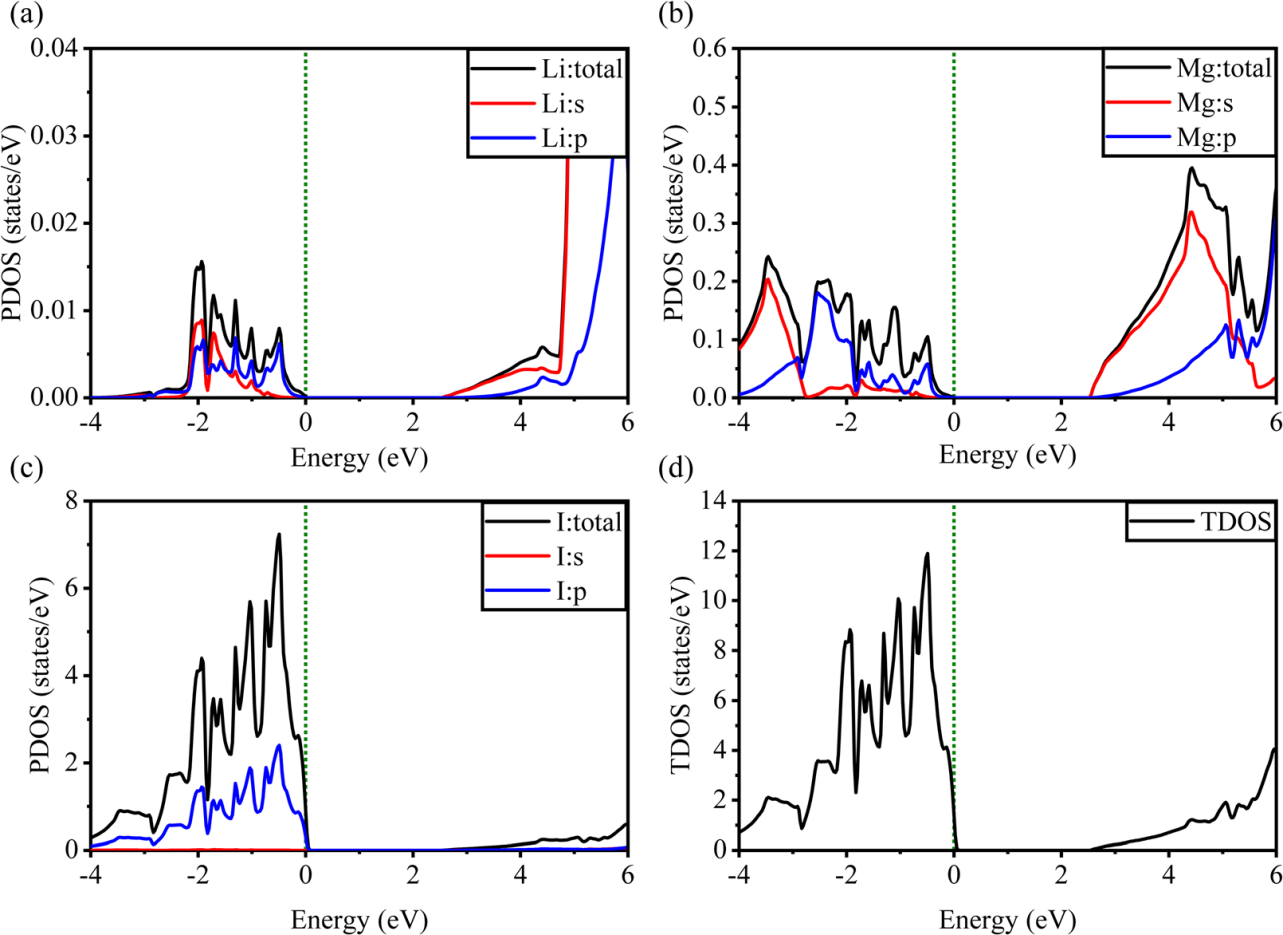
Calculated PDOS of individual atoms in LiMgI_3_ including (a) Li, (b) Mg, and (c) I and (d) TDOS of the LiMgI_3_ perovskite compound.

**Fig. 6:**
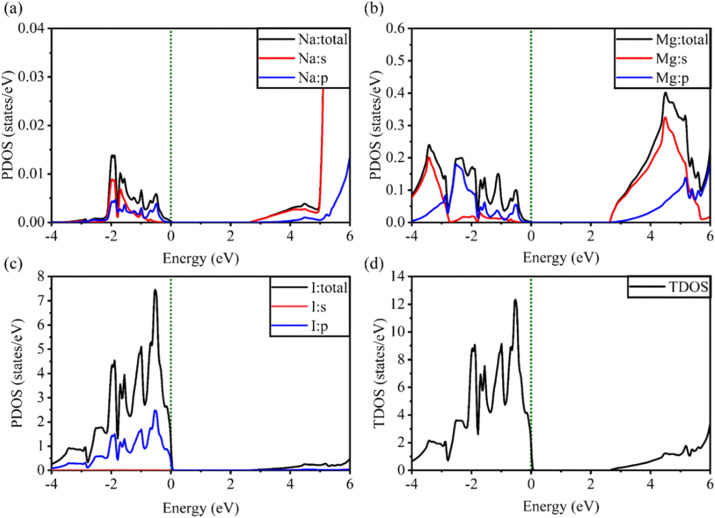
Calculated PDOS of individual atoms in the NaMgI_3_ compound including (a) Na, (b) Mg, and (c) I and (d) TDOS of the NaMgI_3_ perovskite structure.

Fig. 5 and 6 show the analysis of the partial density of states (PDOS) and total partial density of states (TDOS) of the LiMgI_3_ and NaMgI_3_ compounds, respectively. In these figures, the TDOS and PDOS were analyzed to identify their effects on the VB to CB electronic characteristics. Fig. 5a illustrates the dominance of the Li p-state in the VB due to its high intensity near to the Fermi level. The proximity of the peak intensity to the Fermi level is crucial for electron transitions from the VB to the CB. Therefore, the Li p-state is the dominant peak in the electron transfer from VB to CB. In the case of the Mg atom, the p-state contributes to the VB, while the s-state contributes to the CB, as depicted in Fig. 5b. Moreover, Fig. 5c clearly shows that the prominent peak near the Fermi level within the VB primarily originates from the I p-state. In summary, the TDOS in Fig. 5d shows that the VB is mainly contributed by the p-states of Li, Mg, and I, while the s-states of Li and Mg contribute to the CB with a smaller contribution from the I p-states in the LiMgI_3_ compound.

Notably, when replacing the Li atom with Na, similar trends were observed, as depicted in Fig. 6. These similarities arise from the shared properties of Li and Na as elements within the same family. Our investigation is supported by the previous reports on similar families.^20,86^

### 3.6 Optical properties

Details on the optical properties of perovskite compounds can be obtained by calculating various parameters, including the dielectric constant, absorption coefficient, extinction coefficient, refractive index, reflectance, transmission, and loss function. The expression for the complex dielectric function, *ε*(*ω*), with respect to angular frequency is provided as follows:^77–80^

17 *ε*(*ω*) = *ε*_1_(*ω*) + i*ε*_2_(*ω*)

where *ε*_1_(ω) and *ε*_2_(ω) represent the real and imaginary components of the complex dielectric function, respectively, and *ω* is the angular frequency of the incident photon. The absorption coefficient, *α*(*ω*), extinction coefficient, *k*(*ω*), refractive index, *n*(*ω*), reflectivity, *R*(*ω*), conductivity, *σ*(*ω*), and loss function, *L*(*ω*), were calculated based on eqn (17), as follows:^5^18
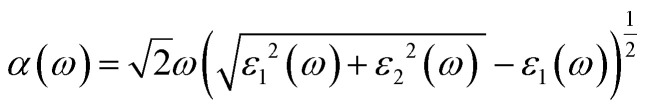
19
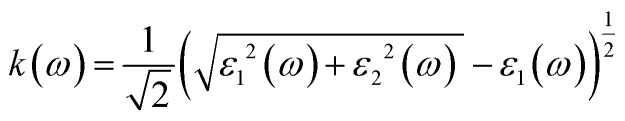
20
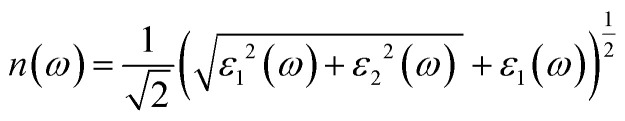
21
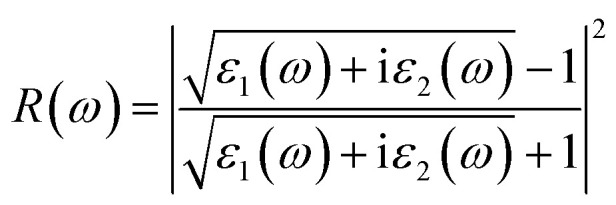
22
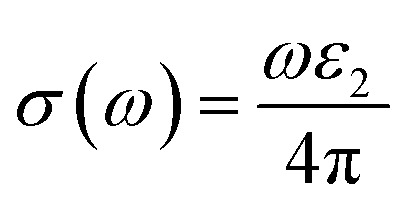
and23
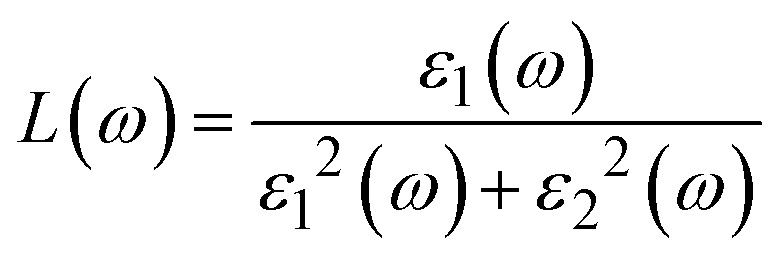


**Fig. 7:**
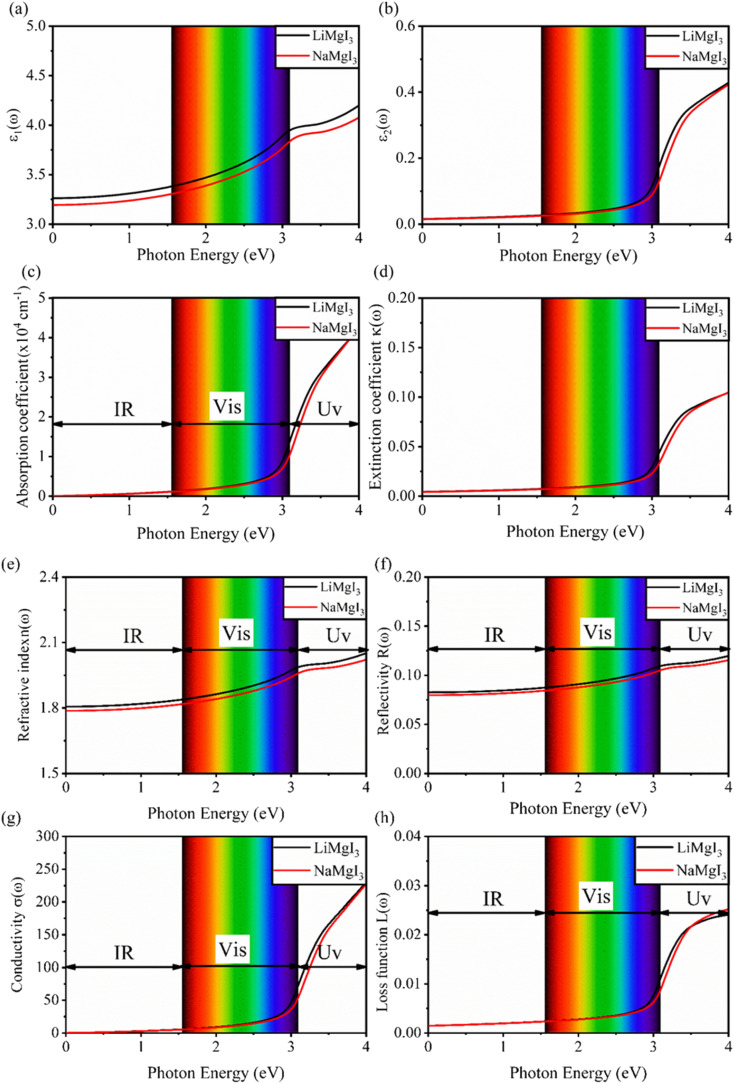
Calculated optical properties of XMgI_3_ (X = Li/Na) alkaline-based single halide perovskite compounds including the dielectric functions for (a) real part (*ε*_1_(*ω*)), (b) imaginary part (*ε*_2_(*ω*)), (c) absorption coefficient (*α*(*ω*)), (d) extinction coefficient (*k*(*ω*)), (e) index of refraction (*n*(*ω*)), (f) reflectivity (*R*(*ω*)), (g) conductivity (*σ*(*ω*)), and (h) loss function (*L*(*ω*)).

Fig. 7 shows the optical properties of the investigated LiMgI_3_ and NaMgI_3_ compounds. Based on the photon radiation on the surface of the compounds, the dielectric function determines their optical properties. Fig. 7a and b show the dielectric functions of the real and imaginary parts of XMgI_3_ (X = Li/Na), respectively. As shown in Fig. 7a, according to the static frequency of *ε*_1_(0), the real dielectric constant is 3.26 for LiMgI_3_ and 3.19 for NaMgI_3_. The *ε*_1_(*ω*) of LiMgI_3_ and NaMgI_3_ reached the maximum peak intensities of 3.96 and 3.86 at the 3.10 eV edge of the visible light region, respectively. However, as shown in Fig. 7b, LiMgI_3_ and NaMgI_3_ revealed 0 eV at *ε*_2_(0), indicating the absence of dissipated energy. The imaginary part of a dielectric material, *ε*_2_(*ω*), increases until energy dissipation occurs in the photon energy absorption region. Thus, the imaginary parts of a dielectric material reflect the behavior of its absorption coefficient.

Fig. 7c shows the absorption coefficient of the cubic structure of LiMgI_3_ and NaMgI_3_. In Fig. 7c, it is evident that both the LiMgI_3_ and NaMgI_3_ compounds initiate photon absorption at 1.3 eV and display a wide absorption peak spanning the infrared (IR) to ultraviolet (UV) region. Therefore, the proposed materials are highly suitable as novel lead-free halide perovskite materials for solar energy harvesting applications. The extinction coefficient demonstrates how the light intensity attenuated on the investigated compounds, LiMgI_3_ and NaMgI_3_, as displayed in Fig. 7d, where both compounds exhibit similar behavior. Thus, the light-absorbing properties of both compounds are similar and consistent with the discussion in Fig. 7c.

Fig. 7e shows the evaluation of the optical characteristics of the refractive index, which explains the bending or refracting behavior of light in a medium. The static refractive indices of LiMgI_3_ and NaMgI_3_ were determined to be 1.81 and 1.78, respectively. This suggests that the square of the refractive index (*n*(*ω*))^2^ corresponds to *ε*_1_(*ω*) at a static frequency, as represented in Fig. 7a. Additionally, both compounds reached their maximum at the edge of higher energy of visible light (3.1 eV). The detection of the refractive index peaks within the low-energy segment of the spectrum is a crucial aspect, especially in the visible region, providing valuable insights for applications in optoelectronic devices. Thus, examining the reflectivity of perovskites offers insights into their surface attributes. Fig. 7f illustrates the reflectance spectra of the LiMgI_3_ and NaMgI_3_ perovskite compounds as a function of photon energy. Both compounds consistently demonstrate low reflectivity (less than 0.10) across the infrared to visible region spectrum, which is negligible. Remarkably, their reflectivity remains consistently low in the visible to infrared range, making these materials well-suited for solar cell applications.

Fig. 7g shows how electromagnetic (EM) radiation induces optical excitation in the compounds, displaying the conductivity *σ*(*ω*) of XMgI_3_ (X = Li and Na). In the case of LiMgI_3_ and NaMgI_3_, their *σ*(*ω*) remains zero in the photon energy range of 0 eV to 0.98 eV due to the absence of optical excitation, indicating the absence of interaction between incident EM radiation and the electrons in these materials in this energy range. As LiMgI_3_ and NaMgI_3_ absorb energy, their electrons shift from the VB to the CB. The consistency of their spectra with the absorption coefficient analysis (Fig. 7c) suggests that absorbing light within the visible part of the electromagnetic spectrum is particularly beneficial for solar cell applications. Another significant consideration involves measuring the energy loss function, denoted as *L*(*ω*), which accounts for inter-band, intra-band, and plasmonic interactions. Additionally, the optical loss serves as an indicator of energy dissipation through scattering, dispersion, and thermal effects, as depicted in Fig. 7h. Fascinatingly, for both LiMgI_3_ and NaMgI_3_, their optical loss in the visible region (1.55–3.10 eV) is nearly insignificant compared to that in the ultraviolet region (3.10–4.0 eV), confirming the suitability of these materials to realize an excellent optoelectronic performance within the visible range of the electromagnetic spectrum.

## 4. Conclusion

This investigation explored the structural, electronic, optical, and mechanical attributes of cubic XMgI_3_ (X = Li/Na) through first-principle calculations by employing CASTEP and the GGA-PBE exchange-correlation function. Our examination of the elastic constants and mechanical properties indicated the notable stability and ductile nature of each perovskite material. Additionally, we investigated the electron charge distribution using Mulliken atomic population analysis and the electron localization function. The developed compounds exhibited advantageous indirect narrow band gaps (2.474 eV for LiMgI_3_ and 2.556 eV for NaMgI_3_) and a broad-spectrum absorption coefficient using the mBJ correlation function in WIEN2k. Based on our findings, especially the band gap and optical properties, these alkali-based halide perovskite materials are suitable for several applications, such as photovoltaics, photocatalysts, and optoelectronic devices. DFT analysis suggested their highly favorable band gap for photovoltaic applications. Consequently, further efforts are required for the synthesis and exploration of these alkali-based halide perovskite materials for realistic applications and future advancements.

## Data availability

This manuscript has associated data in a data repository. The raw/processed data can be made available with a reasonable request to the corresponding author.

## Author contributions

Kefyalew Wagari Guji: conceptualization, methodology, writing-original draft preparation writing-reviewing and editing. Tesfaye Abebe Geleta: conceptualization, methodology, software, writing-reviewing and editing, supervising. Nabil Bouri: methodology, software, writing-reviewing and editing. Victor José Ramirez Rivera: methodology, conceptualization, software, writing-reviewing.

## Conflicts of interest

The authors declare that they have no known competing financial interests.
